# Continuing professional education for general practitioners on chronic obstructive pulmonary disease: feasibility of a blended learning approach in Bangladesh

**DOI:** 10.1186/s12875-020-01270-2

**Published:** 2020-09-28

**Authors:** Md. Nazim Uzzaman, Tracy Jackson, Aftab Uddin, Neneh Rowa-Dewar, Mohammod Jobayer Chisti, G M Monsur Habib, Hilary Pinnock, RESPIRE Collaborators, Harry Campbell, Steve Cunningham, Monica Fletcher, Liz Grant, Sanjay Juvekar, Wong Ping Lee, Andrew Morris, Saturnino Luz, Hana Mahmood, Aziz Sheikh, Colin Simpson, Sajid Bashir Soofi, Osman Yusuf

**Affiliations:** 1 International Centre for Diarrhoeal Disease Research, Bangladesh (icddr,b), Dhaka, Bangladesh; 2 NIHR Global Health Research Unit on Respiratory Health (RESPIRE), Usher Institute, University of Edinburgh, Edinburgh, UK; 3 Usher Institute, University of Edinburgh, Edinburgh, UK; 4 Bangladesh Primary Care Respiratory Society (BPCRS), Khulna, Bangladesh

**Keywords:** Blended learning, COPD, GP, Post-graduate training, Primary care, Continuing medical education, Mixed-methods feasibility study

## Abstract

**Background:**

Continuing medical education (CME) is essential to developing and maintaining high quality primary care. Traditionally, CME is delivered face-to-face, but due to geographical distances, and pressure of work in Bangladesh, general practitioners (GPs) are unable to relocate for several days to attend training. Using chronic obstructive pulmonary disease (COPD) as an exemplar, we aimed to assess the feasibility of blended learning (combination of face-to-face and online) for GPs, and explore trainees’ and trainers’ perspectives towards the blended learning approach.

**Methods:**

We used a mixed-methods design. We trained 49 GPs in two groups via blended (*n* = 25) and traditional face-to-face approach (*n* = 24) and assessed their post-course knowledge and skills. The COPD Physician Practice Assessment Questionnaire (COPD-PPAQ) was administered before and one-month post-course. Verbatim transcriptions of focus group discussions with 18 course attendees and interviews with three course trainers were translated into English and analysed thematically.

**Results:**

Forty GPs completed the course (Blended: 19; Traditional: 21). The knowledge and skills post course, and the improvement in self-reported adherence to COPD guidelines was similar in both groups. Most participants preferred blended learning as it was more convenient than taking time out of their busy work life, and for many the online learning optimised the benefits of the subsequent face-to-face sessions. Suggested improvements included online interactivity with tutors, improved user friendliness of the e-learning platform, and timing face-to-face classes over weekends to avoid time-out of practice.

**Conclusions:**

Quality improvement requires a multifaceted approach, but adequate knowledge and skills are core components. Blended learning is feasible and, with a few caveats, is an acceptable option to GPs in Bangladesh. This is timely, given that online learning with limited face-to-face contact is likely to become the norm in the on-going COVID-19 pandemic.

## Background

Provision of postgraduate training in Family Medicine is increasing in Asia Pacific, but rarely uses innovative online learning [1] that could enhance access to continuing medical education (CME) essential for building and maintaining a high-quality primary care workforce [2]. Traditionally in Bangladesh, post-graduate training involves face-to-face study, but shortage of physicians in many rural and semi-urban areas [3], mean that physicians often cannot leave their practices to attend several days of training. Blended learning is a combination of face-to-face and online learning [4], which has become possible in Bangladesh with recent substantial improvements in internet coverage, and may be a useful way to achieve CME [5].

Chronic obstructive pulmonary disease (COPD) is an exemplar of a condition in which there are concerns that limited awareness of guideline recommendations amongst general practitioners (GPs) [6, 7] leads to misdiagnosis and inappropriate management [8, 9]. COPD affects an estimated 251 million people worldwide [10] and globally, is predicted to be the third leading cause of death by 2030 [11]. Although COPD burden varies between countries, almost 90% of COPD deaths occur in low- and middle-income countries (LMICs) [10]. The national COPD guideline [12] is not widely used in Bangladesh. Some clinicians follow global guidelines [13], however, substantial gaps exist between guideline recommendations and GPs’ practice. Closing this gap is a priority research need for the International Primary Care Respiratory Group (IPCRG) [14].

Blended learning was introduced initially in undergraduate teaching [15–18] and is now extending to postgraduate learning [19], though the concept is relatively new in Bangladesh [20]. An online component allows practitioners increased time and flexibility for study, wider and easier access to learning resources, and a higher level of autonomy in learning than in exclusively face-to-face courses [21, 22]. Management of COPD requires acquisition of practical skills (spirometry; inhaler technique) necessitating a face-to-face component. Therefore, we aimed to assess the feasibility of a blended learning approach to a COPD CME course for GPs in Bangladesh.

## Methods

### Study design

Our mixed-methods feasibility study was conducted in June to August 2019. Quantitative data measured pre-post self-assessment of adherence to COPD guidelines and qualitative focus groups and interviews explored trainee and trainers’ perspectives of the blended learning.

### Inclusion and exclusion criteria

GPs providing public and private primary healthcare services in Bangladesh were invited to participate. GPs in Bangladesh have an MBBS (Bachelor of Medicine and Surgery) are registered by the Bangladesh Medical and Dental Council, have at least two years’ experience of clinical service but with no specialist post-graduate training. We excluded GPs who had previously participated in post-graduate COPD training at any time.

### Participant recruitment

The COPD course, which was provided free of charge, was advertised nationally through the training management portal of the International Centre for Diarrhoeal Disease Research, Bangladesh (icddr,b), and social media was used to disseminate the course advertisement.

Potential participants applied through the icddr,b portal. We screened applicants for eligibility, randomly selected 50 participants who were randomly allocated (using a computer generated randomisation list) to either blended learning or the traditional face-to-face course.

### Sample size

This was a feasibility study, so no sample size calculation was required [23, 24]. Resource availability allowed us to run two courses, so we allocated 25 participants to each group. This is our normal group size, and is a sufficient sample size for assessing feasibility [25].

### Study procedure

The total training hours was 40 h in both blended and traditional learning approaches and the courses contained the same content: components aimed at enhancing COPD knowledge (16 h) and skills (24 h). A private Facebook group was created to provide online learning support for both groups monitored by a tutor and for peer discussion. The tutors were GPs with expertise in respiratory care and had considerable experience of delivering training. The learning approaches are summarised in Table 1 with further details in Additional file 1.

**Table 1:** Blended learning versus traditional learning

	Blended learning	Traditional learning
Learning approach	Online plus classroom-based face-to-face	Only classroom-based face-to-face
Learning hours	40 h: online 16 h; face-to-face 24 h	40 h face-to-face
Learning days	Face-to-face: 3 days (Day 1, Day 23 and Day 24); Online: 21 days (Day 2 to Day 22)	Five consecutive days
Learning support	Private Facebook group during online learning; and face-to-face	Face-to-face; and private Facebook group

### Data collection

#### Quantitative

To assess how the training impacted on participants’ practice and adherence to COPD guidelines, the COPD Physician Practice Assessment Questionnaire (COPD-PPAQ) was administered to all participants prior to starting training and after course completion. Due to Fellowship time restrictions, the COPD-PPAQ was administered only 1 month after the course completed. This validated questionnaire is designed for the self-assessment by physicians of their implementation of 12 key items (two domains: diagnosis and assessment; treatment and follow-up) of COPD guidelines. The answers are globally reproducible [26].

In line with the usual assessment on completion of icddr,b courses, skills were assessed by an oral examination and knowledge was assessed using a written multiple-choice questionnaire examination. Following completion of training, all participants were examined on their COPD knowledge and skills. From previous experience we anticipated that knowledge of COPD and spirometry skills of GPs with no prior COPD training would be very low; we therefore did not assess this pre-training.

#### Qualitative

All participants who completed the blended learning training were invited to participate in one of three focus groups facilitated by MNU supported by a note-taker. Discussion addressed participants’ perceptions of blended learning, preferences compared to previous experiences of face-to-face or online learning, advantages/disadvantages of the blended learning. The three course trainers were interviewed individually to explore their views and opinions about the practicalities of delivering training using this approach (see Additional file 2).

All discussions were digitally recorded and transcribed verbatim in the spoken language (Bengali). The emotional context such as pauses, laughter, emphasis and non-verbal communication were included as notes in the transcripts to aid analysis. Transcripts were translated (by MNU who led the focus groups) from Bengali to English for analysis.

### Analysis

#### Quantitative data

Examination scores, and COPD-PPAQ scores are expressed as percentages. Summary statistics were calculated as means, proportions as necessary. Stata Statistical Software 2015 (StataCorp LP, College Station, Texas, USA) was used for data analysis.

#### Qualitative data

We used thematic analysis for the qualitative data [27] using a coding framework developed by MNU in discussion with the other authors. The focus group discussions with trainees and interviews with trainers were analysed separately. This involved coding the whole data set and the codes were then synthesised into emerging themes which were combined into overarching themes including synthesised data from participants and tutors.

### Reflexivity

The first author is a GP, employed by icddr,b, to deliver CME to healthcare professionals. He was involved in developing the learning materials, and facilitating training sessions which might have influenced the interviews/focus groups and his interpretation of the data. To mitigate against this, themes were discussed within the multi-disciplinary author group.

## Results

We received a total of 637 online applications which were screened for eligibility. The commonest reasons for ineligibility were less than the minimum two years of clinical service (*n* = 227), and already having specialised post-graduate training (*n* = 29). 152 did not provide complete information (eg. No qualification dates or experience) leaving 156 eligible applicants. We randomly selected 50 participants and allocated 25 to each group. Of the allocated participants, 19 (76%) completed blended learning and 21 (84%) traditional learning. The commonest reason for withdrawal in both groups was inability to take time out of practice. Other reasons were illness, domestic or family responsibilities.

### Sociodemographic characteristics of the course attendees

Most of the GPs (63%) were between 26 to 30 years and half had 2–4 years’ experience of patient care. Almost half the participants of both groups were used to consulting with 16 or more patients daily (Table 2).

**Table 2:** Socio-demographics of course attendees

Variable	Blended n (%)	Traditional n (%)
**Age group**		
26–30 years	14 (56.0)	17 (70.8)
31–35 years	5 (20.0)	4 (16.7)
36–40 years	5 (20.0)	2 (8.3)
41–45 years	0 (0)	1 (4.2)
46–50 years	1 (4.0)	0 (0)
**Gender**		
Male	21 (84.0)	21 (87.5)
Female	4 (16.0)	3 (12.5)
**GP experience**		
2–4 years	9 (36.0)	15 (62.5)
5–7 years	9 (36.0)	4 (16.7)
8–10 years	3 (12.0)	2 (8.3)
11+ years	4 (16.0)	3 (12.5)
**Working hours per week**		
< 40 h	8 (32.0)	10 (41.7)
≥ 40 h	17 (68.0)	14 (58.3)
**Number of patients consulted per day**		
4–10	7 (28.0)	9 (37.5)
11–15	5 (20.0)	4 (16.7)
16–20	4 (16.0)	2 (8.3)
21–30	2 (8.0)	3 (12.5)
30+	7 (28.0)	6 (25.0)
**Number of COPD patients seen per month**		
1–10	16 (64.0)	10 (41.7)
11–20	2 (8.0)	4 (16.7)
21–30	3 (12.0)	3 (12.5)
31–50	2 (8.0)	2 (8.3)
51+	2 (8.0)	5 (20.8)
**Ever attended online training previously**		
No	13 (52.0)	15 (62.5)
Yes	12 (48.0)	9 (37.5)

### Quantitative findings

The quantitative results are presented with the caveat that this was a feasibility study which was not powered to show a difference. Detailed outcomes are therefore placed in Additional files 3 and 4 without any statistical comparisons to avoid over interpretation.

#### Comparison of examination scores

The overall end-of-course examination scores was similar in both groups, both for overall knowledge, and for assessment of skills.

#### Self-assessment of practice by the trained GPs

GPs self-reported adherence to COPD guidelines using COPD-PPAQ showed similar improvement in both groups. The self-assessment of 12 key recommendations suggested that participants in both groups scored substantially better in all aspects of their practice except in smoking cessation and referral to specialist.

### Qualitative findings

#### Characteristics of participants

Eighteen of the 19 blended learning course attendees (trainees) who completed the training participated in one of the three focus groups. They were aged 28–50 years and from nine districts of Bangladesh. The location of their workplaces varied from three to over 300 km from the training venue. The number of participants from urban, semi-rural and rural areas were nine, five and four respectively (Table 3). All trainees had previous experience of attending traditional training, half had participated in entirely online training and six had previous experience of a blended learning approach. Interviews were conducted with the three trainers who were between 44 and 64 years of age. No further details are provided to maintain confidentiality of trainers.

**Table 3:** Summary of socio-demographic characteristics of focus group participants

Identifier	Age (year)	Sex	Geographical area	Distance from training venue (km)
FGD1				
P1	38–42	Male	Urban	0–50
P2	28–32	Male	Urban	0–50
P3	28–32	Male	Semi-rural	101–150
P4	28–32	Male	Rural	201–250
P5	38–42	Male	Rural	201–250
P6	33–37	Male	Urban	0–50
FGD2				
P7	33–37	Male	Semi-rural	51–100
P8	28–32	Male	Urban	0–50
P9	28–32	Male	Urban	0–50
P10	33–37	Male	Rural	201–250
P11	28–32	Male	Semi-rural	0–50
P12	33–37	Male	Urban	301–350
FGD3				
P13	28–32	Female	Urban	0–50
P14	28–32	Female	Urban	0–50
P15	33–37	Male	Urban	0–50
P16	28–32	Male	Rural	101–150
P17	48–52	Male	Semi-rural	0–50
P18	28–32	Male	Semi-rural	0–50

#### Summary of themes

Three main themes emerged in the analysis of both focus group discussions with trainees and interviews with course trainers. The themes and sub-themes are listed in Table 4 and described below.

**Table 4:** Themes and sub-themes

Themes	Sub-themes
Theme I: Convenience and flexibility	Preference of learning approach
	Confidence of trainees
Theme II: Educational advantages and disadvantages	Advantages of blended learning
	Disadvantages of blended learning
Theme III: Suggestions for improvement	E-Learning module
	Receiving feedback online
	Technological issues
	Practical session
	Face-to-face class
	Financial

### Theme I: convenience and flexibility

#### Preference for learning approach

Most participants thought that blended learning was a better and more convenient mode of training compared to either traditional or online training.

*“This* [blended learning] *approach is very new to our country. I didn’t know anything about it previously. One of my acquaintances suggested me to apply for this training and I did it without thinking much. When I was selected for the training, I was in doubt whether I could continue it from* [a remote location] *leaving my job for several long days as I saw that the total duration of course was about four weeks*! *When I attended the orientation class, I found the blended learning model to be very convenient for me as I don’t have to stay here for very long”.* (Trainee, P10)

This was echoed by the trainers who were positive about the online resources being available *‘24 hours-anytime, anywhere’*.

In contrast, one trainee preferred the traditional approach because it enabled him to focus on the topic for the duration of the course, whereas online learning could too easily be postponed. He also considered that the traditional approach was better for practical demonstrations (e.g. cardio-pulmonary resuscitation).

*“Well* […], *I’ll choose traditional approach because in only face-to-face training, we have a mindset and allocate a dedicated time only for learning. Here (in blended approach), what happened to me, I thought that I’d read now but it did not happen thinking that I could learn it later because of flexibility”.* (Trainee, P15)

One of the course trainers preferred the traditional approach, although he recognised that it was difficult for busy GPs to be away from their practice.

#### Confidence of trainees

Almost all the trainees felt confident of their knowledge and skills in diagnosing and managing COPD patients after completing the training. Most wished to participate in future courses using a blended approach and said they would recommend it to others. One participant was sufficiently confident in his acquired knowledge and skills that he felt he would be able to disseminate what he had learnt to staff in his practice.

*“Regarding knowledge, I’ll say that now there is sky-land* [very far] *distance from my baseline knowledge! My views and skills have been changed a lot. Previously, I couldn’t use or advise inhaler devices properly to patients. Now I really feel confident and I can also help my hospital nurses to improve their knowledge in this regard”.* (Trainee, P10)

Most of the trainees felt confident about interpreting spirometry findings, an essential practical component of the course, delivered mostly face-to-face with some components online.

*“Yesterday, I advised a patient to perform spirometry. Previously I used to see comments in a tracing, although I work in a large specialised hospital* [!]*. This time, I interpreted it well and diagnosed the patient accordingly which gave me immense pleasure”.* (Trainee, P15)

In contrast, a few participants felt that they did not get enough time to perform spirometry manoeuvres during the face-to-face sessions (though this was the same as in the traditional course).

*“During practical session I expected more to learn about spirometer (how to operate the machine). However, we didn’t have the scope to learn spirometer, especially with real patient”.* (Trainee*,* P4)

### Theme II: educational advantages and disadvantages

#### Advantages of blended learning

Reasons provided for preferring the blended learning approach were the convenience of not having to relocate and the option to do some of the training in their own time which fitted around their practice work. Reducing their physical presence in class was considered very helpful as it caused minimal interruption to their patient care. This view was particularly apparent in accounts from doctors who worked in rural areas and remote places where learning opportunities are limited, and staff resource is at a critically low level.

*“Those of us who live in remote areas; the blended approach is a blessing for us which would allow us to add to our knowledge deficit quite a lot. Those who stay centrally, get many opportunities to attend scientific seminars, CME (continued medical education) etc which we couldn’t manage”.* (Trainee, P4)

In the blended learning approach, participants learned online before they attended face-to-face classes when they could solve the queries that had arisen while using the online resources.

*“We got learning contents in advance and were able to go through online. We know in advance what we will learn tomorrow. We solved our queries that arose during online learning when we were in face-to-face classes.”* (Trainee, P12)

A few participants said that blended was more attractive and interactive compared to a traditional approach or only online training. Two of the trainers mentioned that the blended approach offered two-way learning with scope for providing better student support compared to either traditional or entirely online training.

#### Disadvantages of blended learning

Most of the trainees did not mention any generic drawbacks of the blended-learning approach. Instead they discussed the weakness of the particular e-learning module they had used, and highlighted a few areas of the face-to-face classes which needed improving. Some trainees found reading online content uncomfortable, mentioning that they were more comfortable with familiar paper rather than online documents. Specifically, excessive screen exposure caused eye pain and headache to one of the trainees. Although most participants completed the online module, a few mentioned that they had neglected the online learning either deliberately thinking that they would learn it from the face-to-face classes or procrastinating and not quite getting round to doing it in their busy schedules.

*“Because of having the face-to-face part, we often have neglected the online part thinking that we have face-to-face classes”!* (Trainee, P18)

Apart from sharing the concern about the discomfort of online reading, trainers had some additional concerns about blended learning. One trainer was concerned that the online component might be considered as an extra pressure by some trainees. Another trainer thought that the three-week gap between the online and face-to-face learning might increase participant dropout from the course. In addition, one of the trainers noted that unreliable internet access in some locations might limit the usefulness of the blended approach in Bangladesh. In addition, this trainer was concerned that many physicians were not accustomed to using computers and if they only completed the minimum face-to-face tasks it might affect skill development of the trainees.

### Theme III: suggestions for improvement

#### E-learning module

Almost all participants (trainees and trainers) thought the e-learning module needed further development, with suggestions about more videos, animation, and quizzes with analytical questions to make it more interactive and attractive.

*“Regarding online content, I’d say there are many areas for improvement. Say, you could add videos, animations, quiz etc”. (Trainee, P3)*

Opinions were divided about whether the contents were *‘somewhat disorganised’*. Some trainees suggested including the content of the subsequent face-to-face classes in the e-learning module so that learning was reinforced.

*“Practical sessions like inhaler techniques may be given online which would help us to learn better as we may not learn the technique in one face-to-face class. In future, if we get confused, we can watch the video and make our technique correct”. (Trainee, P13)*

#### Receiving feedback online

Most of the trainees wanted prompt feedback via the online platform rather than having to use a separate Facebook group for this purpose. Facebook was associated with social communication during leisure time and not as an effective medium for solving professional queries. Indeed, some people noted that it was a distraction which wasted a lot of time. Moreover, participants had only met once during the orientation class, so some did not feel sufficiently familiar with each other to be able to engage proactively in online group discussions. From a practical perspective they had to open Facebook separately alongside the e-learning module which they found burdensome and although 14 delegates tried it at least once, only 5 delegates engaged in discussion.

*“Yes, we had a Facebook group* [for solving queries]*. But to me, when I logged on it, a lot of time went away unknowingly.” (Trainee, P15)*

A few trainees said that provision of a tutor for a scheduled online discussion would be helpful to solve queries and this would allow more time for practical tasks during face-to-face classes. Two of the trainers with previous experience of online discussions, agreed and considered that the online discussion could help trainees to engage and learn more.

*“The provision of online discussion would help participants to learn more. Even participants could ask question online which they couldn’t understand in face-to-face classes”. (Trainee, P3)*

In contrast, some trainees considered that a fixed time for an online discussion was unlikely to be convenient for everyone, and reduced the flexibility that was an advantage of the online learning. They suggested that face-to-face classes were a better option for solving their queries.

*“I don’t think we can align our time with the online tutor”. (Trainee, P4)*

*“Since we had the opportunity of face-to-face classes, here we didn’t have the need of online classes”. (Trainee, P5)*

Other trainees suggested that the e-learning platform should have a discussion board where a mentor would give his/her feedback, and everyone could see answers and learn accordingly.

*“I’d say that the online platform itself should keep an option of asking question* […] *a coordinator will reply to our queries in a particular time of a day”. (Trainee, P8)*

#### Technological issues

The majority of the trainees encountered challenges reading the online contents; only two participants did not have any problems. There were difficulties reading documents in full screen, sometimes a chapter showed as ‘incomplete’ even though it had been completed. A few trainees with previous experience of online courses suggested that chapters should be completed in order to qualify for the chapter accomplishment quiz. One trainee wanted the option of a mobile-based application along with the provision of offline access to the contents that they completed earlier for rereading as necessary.

*“Mobile based app could be introduced where we can even get access without having internet connection [smiling]”. (Trainee, P9)*

#### Practical session

Almost all trainees shared that the practical sessions should involve “patients”, if only for a short period of time.

*“[…] The practical classes were mostly device oriented. Since we will apply our knowledge on patients, I think the practical classes need to be real patient-based which will make the course much more effective”. (Trainee, P1)*

#### Face-to-face class

The majority of the trainees thought that face-to-face classes would be more convenient if they were delivered 7 days apart, preferably during weekends, so they would not need to leave their practice during working days.

*“[…] We all are busy, or it is difficult to manage leave for two-three consecutive days for face-to-face training. Classes could be taken seven days apart and during weekend”. (Trainee, P3)*

#### Financial

Two of the participants wanted an honorarium for participating in the course while one participant strongly opposed this issue.

*“We have been provided with food during training. If you could provide us some honorarium, that would be very good. After a certain age, we have more financial liabilities.” (Trainee, P9)*

In contrast, one trainer was concerned about the non-attendance of some participants suggesting that a course fee should be paid by the participants to make them more responsible.

*“This time we found that few participants didn’t complete the course although there were many applicants who were very interested to attend the course.* […..] *One of the reasons might be that the participants didn’t have to pay the course fee of their own”. (Trainer, T3)*

## Discussion

### Summary of findings

Of the 25 trainees allocated to each group, 19 completed blended learning and 21 completed the traditional face-to-face learning. Inability to take time-out of practice was the commonest reason for attrition in both groups. The gain in knowledge and skills by the participants in both groups was similar. In addition, self-reported adherence to COPD guidelines before and after training revealed similar improvement in both groups. All participants, except one trainer and one trainee, preferred the blended learning approach as it was more convenient within their busy work schedules. Although a few participants ‘neglected’ the online modules, for most the online learning optimised the benefits of the face-to-face sessions. There were a number of practical problems with internet connections and finding it ‘uncomfortable’ to read on-screen documents and most participants suggested improving interactivity. Online support from tutors was valued, but embedded in the learning platform rather than using Facebook which was associated with social interaction.

### Strengths and limitations

A strength of our mixed method design is that it allowed triangulation of results; for example, the participants’ perception of increased confidence in managing COPD was matched by measured gains in skills and knowledge. The quantitative data will inform potential outcomes for a future evaluation of blended learning on COPD and the qualitative data gave insights into both positive and negative perspectives. Moreover, the practical suggestions and operational challenges will be helpful in refining future training.

The examiners were aware of the allocation of both groups which risked biasing the quantitative outcomes, but the same examiners assessed participants from both groups ensuring consistency of assessment. Blinded assessment was not possible within the resources of the study. We were aware of the impact of reflexivity, as the researcher conducting the focus groups and interviews (MNU) was also involved with training coordination and development of learning materials. Involvement of a multidisciplinary author group unconnected with icddr,b or the course helped ensure a balanced interpretation of the data. Although we achieved data saturation with respect to the trainee opinions, the limited number of trainers meant we only heard three perspectives. Our aim was to assess the feasibility of the blended learning intervention, and the single location (Dhaka) of the course and the small numbers limit generalisable, though our findings may be applicable to others working in similar settings in Bangladesh or beyond.

### Interpretation in the light of published literatures

#### Perception of blended learning

Studies show that blended learning allows greater flexibility and responsiveness in adult learning processes [16, 28, 29]. The addition of online learning overcomes limitations of time and space, reaches more students and supports instructional methods that may be hard to achieve without increased resources [30]. Some studies have found a mismatch regarding preferred learning approaches where trainers assumed that technology-based learning suited the trainees’ style; however, trainees felt differently [5]. In our study, trainees and trainers almost all agreed that blended learning overcame two limitations compared to entirely online or traditional learning. First, the e-learning component reduced the need for prolonged time out of practice to attend a course, and second, the prior online work optimised the learning of skills in the face-to-face class. A previous study with GPs also found e-learning a useful way to gain knowledge and the face-to-face component a suitable way of transferring practical knowledge [31]. Furthermore, some participants in our study suggested blended learning was cost-effective [32] as a substantial number of doctors could be trained within a short period of time [33].

In contrast, a few trainees found it difficult to adapt their learning styles to a blended approach [5]. Some felt that provision of paper versions of the e-learning module would be helpful as they were accustomed to reading paper books [31]. Flexibility is generally seen as a strength as e-learning allows participants to learn at a convenient time [34]. In our study, it was also viewed as a challenge because some GPs found it difficult to schedule study time. Also, some neglected online study hoping to catch-up in the face-to-face sessions. Trainers living at a distance found it less efficient to schedule face-to-face classes involving long travel time for shorter meetings.

#### Operational challenges

The lack of a blended learning approach to CME in LMICs may be associated with limited technological resources [35]. Echoing other studies that have highlighted poor access to technology as a barrier to the implementation of technology-enhanced teaching [36], our participants described annoying technical problems such as losing information on progress, or the need to switch between pages. The use of social media (in particular Facebook) was associated with social communication and considered an ineffective way of interacting with fellow participants and solving queries though other studies have successfully used this approach [37]. Like several other studies, some trainees and trainers considered that, for productive interaction, it is important that tutors actively moderate online discussions [38–40]. More patient involvement in skills development was wanted, and contributing to online modules could be a convenient way to incorporate patients.

The dropout of participants (1 in 5 in our study) was another challenge. An outbreak of dengue fever in Bangladesh was one factor and cultural context is also important. In LMICs like Bangladesh, food and honorarium are two important issues that need to be considered when developing education. Government employees typically expect to receive an honorarium when they participate in any training.

### Implications for clinical education and research

Provision of accessible CME is central to maintaining the quality of primary healthcare and the morale of the workforce [2]. In the context of COPD, where under-diagnosis and inadequate management is common [41–43], our blended-learning course was a feasible approach to enhancing knowledge and skills of GPs about COPD. The observation by some of the participants that they were sufficiently confident in their learning to be able to pass on the knowledge to others in their practices is encouraging but needs further evaluation. ‘Train the Trainer’ programmes have been used successfully by the International Primary Care Respiratory Group [44], and blended learning offers the potential for online modules to be used to pass on knowledge. The flexible and practical blending of online and face-to-face learning has the potential to be used for CME of other long-term conditions in Bangladesh and beyond.

## Conclusions

With some caveats, blended learning was an acceptable educational model and preferred by most of the busy GPs in Bangladesh. Quality improvement requires a multifaceted approach, but adequate knowledge and skills are a core component; blended learning is a feasible option which could contribute to improved implementation of guideline recommendations. Online CME was a novel approach in our LMIC setting, but learning with limited face-to-face contact is likely to become the norm in the current COVID-19 pandemic making this a timely message.

## Supplementary information

**Additional file 1.** Programme outline.

**Additional file 2.** Topic guide for focus group discussion and interview.

**Additional file 3.** Comparison of scores.

**Additional file 4.** Practice assessment of trained physicians using COPD Physician’s Practice Assessment Questionnaire (COPD-PPAQ).

## Data Availability

The qualitative data that support study findings may be available from the corresponding author on request.

## Abbreviations

CME: Continuing Medical Education
COPD: Chronic obstructive pulmonary disease
COPD-PPAQ: COPD Physician practice assessment questionnaire
GP: General practitioner
icddr,b: International Centre for Diarrhoeal Disease Research, Bangladesh
IPCRG: International Primary Care Respiratory Group
LMICs: Low- and middle-income countries
MBBS: Bachelor of Medicine and Surgery

## References

[1] Wong CH, Phoa S (2016). The status of family medicine training programs in the Asia Pacific. Fam Med.

[2] Lee KP, Wong C, Chan D, Kung K, Luk L, Wong MCS (2019). Family medicine vocational training and career satisfaction in Hong Kong. BMC Fam Pract.

[3] Ahmed SM, Hossain MA, Rajachowdhury AM, Bhuiya AU (2011). The health workforce crisis in Bangladesh: shortage, inappropriate skill-mix and inequitable distribution. Hum Resour Health.

[4] Watson J. Blended learning: the convergence of online and face-to-face education. Promising Practices in Online Learning. North American Council for Online Learning. 2008. https://files.eric.ed.gov/fulltext/ED509636.pdf. Accessed 7 June 2020.

[5] Salim H, Lee PY, Ghazali SS, Ching SM, Ali H, Shamsuddin NH (2018). Perceptions toward a pilot project on blended learning in Malaysian family medicine postgraduate training: a qualitative study. BMC Med Educ..

[6] Sandelowsky H, Krakau I, Modin S, Ställberg B, Nager A (2019). COPD patients need more information about self-management: a cross-sectional study in Swedish primary care. Scand J Prim Health Car.

[7] Bertella E, Zadra A, Vitacca M (2013). COPD management in primary care: is an educational plan for GPs useful?. Multidiscip Resp Med..

[8] Ragaišienė G, Kibarskytė R, Gauronskaitė R, Giedraitytė M, Dapšauskaitė A, Kasiulevičius V (2019). Diagnosing COPD in primary care: what has real life practice got to do with guidelines?. Multidiscip Resp Med.

[9] Ho T, Cusack RP, Chaudhary N, Satia I, Kurmi OP (2019). Under- and over-diagnosis of COPD: a global perspective. Breathe (Sheff).

[10] World Health Organisation (WHO). Chronic obstructive pulmonary disease (COPD): Key facts. 2017. https://www.who.int/en/news-room/fact-sheets/detail/chronic-obstructive-pulmonary-disease-(copd). Accessed 7 Jun 2020.

[11] Mathers CD, Loncar D (2006). Projections of global mortality and burden of disease from 2002 to 2030. PLoS Med.

[12] Asthma Association Bangladesh. National Guidelines: Asthma, Bronichiolitis, and COPD. 2005. https://www.scribd.com/document/350879943/National-Asthma-Bronchiolitis-COPD-Guidelines. Accessed 7 Jun 2020.

[13] Global Initiative for Chronic Obstructive Lung Disease (GOLD). Global Strategy for the Diagnosis, Management, and Prevention of Chronic Obstructive Pulmonary Disease. 2020. https://goldcopd.org/wp-content/uploads/2019/11/GOLD-2020-REPORT-ver1.0wms.pdf. Accessed 7 Jun 2020.

[14] Pinnock H, Thomas M, Tsiligianni I, Lisspers K, Østrem A, Ställberg B (2010). The international primary care respiratory group (IPCRG) research needs statement 2010. Prim Care Respir J.

[15] Bösner S, Pickert J, Stibane T (2015). Teaching differential diagnosis in primary care using an inverted classroom approach: student satisfaction and gain in skills and knowledge. BMC Med Educ..

[16] Lewin LO, Singh M, Bateman BL, Glover PB (2009). Improving education in primary care: development of an online curriculum using the blended learning model. BMC Med Educ..

[17] Edginton A, Holbrook J. A blended learning approach to teaching basic pharmacokinetics and the significance of face-to-face interaction. Am J Pharm Educ. 2010;74(5):88. 10.5688/aj740588 PMC2907853 20798797

[18] De Leng BA, Dolmans DH, Muijtjens AM, Van Der Vleuten CP (2006). Student perceptions of a virtual learning environment for a problem-based learning undergraduate medical curriculum. Med Educ.

[19] Westerlaken M, Christiaans-Dingelhoff I, Filius RM, de Vries B, de Bruijne M, van Dam M (2019). Blended learning for postgraduates; an interactive experience. BMC Med Educ.

[20] Uzzaman MN, Banu S, Habib GM, Hossain AE, Kabir MJ, Karim MR (2018). Improving Physicians’ capacity for chronic obstructive pulmonary disease care through blended E-learning: a pilot study in Bangladesh. Cureus..

[21] Güzer B, Caner H (2014). The past, present and future of blended learning: an in depth analysis of literature. Procedia Soc Behav Sci.

[22] Ellaway R, Masters K (2008). AMEE guide 32: e-learning in medical education part 1: learning, teaching and assessment. Med Teach.

[23] Tickle-Degnen L (2013). Nuts and bolts of conducting feasibility studies. Am J Occup Ther.

[24] National Institute for Health Reseach. Information for authors: Pilot and Feasibility Studies. https://www.journalslibrary.nihr.ac.uk/information-for-authors/pilot-and-feasibility-studies/. Accessed 7 Jun 2020.

[25] Billingham SA, Whitehead AL, Julious SA (2013). An audit of sample sizes for pilot and feasibility trials being undertaken in the United Kingdom registered in the United Kingdom clinical research network database. BMC Med Res Methodol.

[26] Boulet LP, Devlin H, O'Donnell DE (2011). The Physicians’ practice assessment questionnaire on asthma and COPD. Respir Med.

[27] Braun V, Clarke V (2006). Using thematic analysis in psychology. Qual Res Psychol.

[28] Lotrecchiano GR, McDonald P, Lyons L, Long T, Zajicek-Farber M (2013). Blended learning: strengths, challenges, and lessons learned in an interprofessional training program. Matern Child Health J.

[29] Bonk CJ, Olson TM, Wisher RA, Orvis KL. Learning from focus groups: an examination of blended learning. International Journal of E-Learning & Distance Education/Revue internationale du e-learning et la formation à distance 2002;17(3):97–118.

[30] Gray K, Tobin J (2010). Introducing an online community into a clinical education setting: a pilot study of student and staff engagement and outcomes using blended learning. BMC Med Educ..

[31] Te Pas E, Meinema JG, Visser MR, van Dijk N (2016). Blended learning in CME: the perception of GP trainers. Educ Prim Care.

[32] Lothridge K, Fox J, Fynan E (2013). Blended learning: efficient, timely and cost effective. Aust J Forensic Sci.

[33] Singh H (2003). Building effective blended learning programs. Educ Technol.

[34] Harden RM (2005). A new vision for distance learning and continuing medical education. J Contin Educ Heal Prof.

[35] Rowe M, Frantz J, Bozalek V (2012). The role of blended learning in the clinical education of healthcare students: a systematic review. Med Teach..

[36] Czerniewicz L, Brown C (2005). The uses of information and communication (ICT) in teaching and learning in south African higher education practices in the Western cape : research : information and communication technologies. Perspect Educ.

[37] Siddiqui M, Bukhari AS, Shamael I, Shah ZA, Maken N (2018). Facebook as a learning tool: perception of stroke unit nurses in a tertiary care hospital in Islamabad. Cureus..

[38] Wilson T, Whitelock D (1998). What are the perceived benefits of participating in a computer-mediated communication (CMC) environment for distance learning computer science students?. Comput Educ.

[39] Alebaikan R, Troudi S (2010). Online discussion in blended courses at Saudi universities. Procedia Soc Behav Sci.

[40] Lord G, Lomicka L (2008). Blended learning in teacher education: an investigation across media. Contemp Issues Technol Teach Educ.

[41] Rabe KF (2007). Global initiative for chronic obstructive lung disease. Global strategy for the diagnosis, management, and prevention of chronic obstructive pulmonary disease. GOLD executive summary. Am J Respir Crit Care Med.

[42] Lindberg A, Bjerg A, Ronmark E, Larsson LG, Lundback B (2006). Prevalence and underdiagnosis of COPD by disease severity and the attributable fraction of smoking Report from the obstructive lung disease in northern Sweden studies. Respir Med.

[43] Stallberg B, Janson C, Johansson G, Larsson K, Stratelis G, Telg G (2014). Management, morbidity and mortality of COPD during an 11-year period: an observational retrospective epidemiological register study in Sweden (PATHOS). Prim Care Respir J..

[44] McDonnell J, Williams S, Ryan D, Pinnock H, de Sousa JC (2018). Improving care for people with asthma: building capacity across a European network of primary care organisations–the IPCRG’s teach the teacher Programme. J Glob Health Rep.

