# Supplementary material for: Continuing professional education for general practitioners on chronic obstructive pulmonary disease: feasibility of a blended learning approach in Bangladesh
Source: BMC Fam Pract. 2020 Sep 28;21:203. doi: 10.1186/s12875-020-01270-2 (PMC7521769; doi:10.1186/s12875-020-01270-2)
Supplement: Supplementary file 1 — Additional file 1. Programme outline. [file 12875_2020_1270_MOESM1_ESM.docx]

**Additional file 1:** Programme outline

| **Blended learning** |
| --- |
| **Day 1 (9 am to 5 pm): Face-to-face**   - Orientation and access to COPD e-learning module - Entry to learning group - Overview of the COPD module |
| **Day 2-22: Online learning**  Self-paced e-learning of COPD module |
| **Day 23 (9 am to 5 pm): Face-to-face**   - Practical session with inhalers devices - Basics of spirometry |
| **Day 24 (9 am to 5 pm): Face-to-face**   - Spirometry practical session |
| **Traditional learning** |
| **Day 1 (9 am to 5 pm)**   - Definition and overview of COPD - Pathology, pathogenesis and pathophysiology - Diagnosis and assessment (part 1) |
| **Day 2: (9 am to 5 pm)**   - Diagnosis and assessment (part 2) - Drugs used in COPD - Practical session with inhalers devices |
| **Day 3: (9 am to 5 pm)**   - Basics of spirometry - Management of stable COP |
| **Day 4: (9 am to 5 pm)**   - Spirometry practical session - Management of exacerbations |
| **Day 5: (9 am to 5 pm)**   - Pulmonary rehabilitation - Organizational care - Supportive, palliative, end-of-life & hospice care |
