# Supplementary material for: Continuing professional education for general practitioners on chronic obstructive pulmonary disease: feasibility of a blended learning approach in Bangladesh
Source: BMC Fam Pract. 2020 Sep 28;21:203. doi: 10.1186/s12875-020-01270-2 (PMC7521769; doi:10.1186/s12875-020-01270-2)
Supplement: Supplementary file 2 — Additional file 2. Topic guide for focus group discussion and interview. [file 12875_2020_1270_MOESM2_ESM.docx]

**Additional file 2:** Topic guide for focus group discussion and interview

**Preamble:**

- Ice-breaking and explain that there is no right or wrong answer
- Explain need to get consent for the focus group discussion/interview and audio-recording.
- Explain that the participant does not have to answer if he or she does not wish to do so and obtain consent.

**Trainee (focus group)**

| **Topic** | **Question** | **Prompts** |
| --- | --- | --- |
| Perception on blended learning | 1. What do you think about the blended learning approach?  2. How do you compare this training with your previous experience of traditional training? | - Your feelings - Advantages and - Disadvantages of blended learning |
| Confidence after training | 1. Did you feel more/less confident with what you had learnt face-to-face or online– and why? | - Knowledge - Skills - Dealing with COPD patients - Motivations |
| Online learning experience | 1. Was it easier/more difficult to ask questions and get feedback– and why?  2. Had you faced any challenges?  3. How did you overcome? | - User friendliness - e-learning module - Use of Facebook - Challenges overcome |
| Face-to-face classes | 1. What do you think about the face-to-face classes? 2. 2. Had you faced any challenges? 3. How did you overcome? | - Practical sessions - Time management - Spirometry sessions - Challenges overcome |
| Recommendation for future courses | 1. What changes would you recommend for future rehabilitation? 2. Will you participate in another blended learning course based on this experience? | - Duration - Settings - Frequency - Distance from centre |

**Trainer (interview)**

| **Topic** | **Question** | **Prompts** |
| --- | --- | --- |
| Perception on blended learning | 1. What do you think about the blended learning approach?  2. How do you compare this training with your previous experience of traditional training? | - Your feelings - Advantages and - Disadvantages of blended learning |
| Effectiveness as a training approach | 1.Did you feel the blended learning teaching was as more/less effective as face-to-face – and why? | - Time, duration - Logistic support - e-learning module - Student support - Practical session |
| Recommendation for future courses | 1. What changes would you recommend for future courses? | - Duration - Settings - Frequency - Distance from centre |
