# Supplementary material for: Continuing professional education for general practitioners on chronic obstructive pulmonary disease: feasibility of a blended learning approach in Bangladesh
Source: BMC Fam Pract. 2020 Sep 28;21:203. doi: 10.1186/s12875-020-01270-2 (PMC7521769; doi:10.1186/s12875-020-01270-2)
Supplement: Supplementary file 3 — Additional file 3. Comparison of scores. [file 12875_2020_1270_MOESM3_ESM.docx]

**Additional file 3:** Comparison of scores

| **Overall score** | **Blended (n=19)** | | **Traditional (n=21)** | |
| --- | --- | --- | --- | --- |
| Knowledge assessment | 91.2% | | 89.3% | |
| Skill assessment | 83.3% | | 75.6% | |
| Practice assessment | Before | After | Before | After |
|  | 41.2% | 73% | 44.1% | 69.9% |
