# Supplementary material for: Continuing professional education for general practitioners on chronic obstructive pulmonary disease: feasibility of a blended learning approach in Bangladesh
Source: BMC Fam Pract. 2020 Sep 28;21:203. doi: 10.1186/s12875-020-01270-2 (PMC7521769; doi:10.1186/s12875-020-01270-2)
Supplement: Supplementary file 4 — Additional file 4. Practice assessment of trained physicians using COPD Physician’s Practice Assessment Questionnaire (COPD-PPAQ). [file 12875_2020_1270_MOESM4_ESM.docx]

**Additional file 4:** Practice assessment of trained physicians using COPD Physician’s Practice Assessment Questionnaire (COPD-PPAQ)

| **Indicators** | **Blended (n=19)** | | **Traditional (n=21)** | |
| --- | --- | --- | --- | --- |
|  | Before | After | Before | After |
| Confirm diagnosis by pulmonary function tests | 15.1 | 57.9 | 30 | 61.3 |
| Assess level of function and disability | 10.5 | 80.6 | 18.3 | 76.7 |
| Document frequency and severity of COPD exacerbations | 22.9 | 72.4 | 20.6 | 67.1 |
| Prescribe at least one long-acting bronchodilator | 49.0 | 85.5 | 46.7 | 72.9 |
| Prescribe inhaled corticosteroid (ICS) + long-acting beta 2 -agonist (LABA) in combination and tiotropium if MRC >3 + exacerbations (>1/year) | 37.1 | 68.7 | 49.3 | 72.4 |
| Provide an exercise prescription to promote regular physical activity | 32.9 | 70.3 | 39.8 | 75.7 |
| Refer to pulmonary rehabilitation program if >MRC 3 | 25.0 | 66.4 | 27.4 | 40.9 |
| Provide written referral for structured patient education | 29.2 | 59.2 | 30.2 | 59.5 |
| Provide a written action plan for exacerbation management | 38.1 | 67.4 | 30.5 | 57.1 |
| Provide smoking cessation counselling and pharmacological intervention if smoking | 88.9 | 86.1 | 82.8 | 85.2 |
| Assess inhaler/device technique (or refer to COPD educator) at each visit | 70.5 | 87.6 | 73.3 | 93.3 |
| Refer to specialist if diagnosis is uncertain, if clinical deterioration is rapidor if home oxygen or surgical options are being considered | 75.3 | 75.5 | 79.3 | 76.7 |
| Overall score* (mean) | 41.2 | 73.0 | 44.1 | 69.9 |

*Units are scores out of a total of 100
